# What is the role of video feedback in supporting parents experiencing mental health problems?

**DOI:** 10.1192/j.eurpsy.2021.78

**Published:** 2021-08-13

**Authors:** J. Barlow

**Affiliations:** Department Of Social Policy And Intervention, University of Oxford, Oxford, United Kingdom

## Abstract

**Abstract Body:**

Parental mental health problems have been found to have a significant impact on a range of aspects of parental caregiving during the postnatal period, with significant implications in terms of key aspects of the child’s development. Video feedback is a generic term that refers to the use of videotaped interactions of the parent and child to promote parental sensitivity, and a recent meta‐analysis of 20 studies (1757 parent‐child dyads) found that video feedback can improve parental sensitivity compared with a control or no intervention up to six months’ follow‐up. This paper will examine the ways in which video feedback might contribute to the ability of parents with mental health problems to provide the type of caregiving that will promote the development of a secure attachment in the infant.

**Disclosure:**

No significant relationships.

